# Canadian Resources, Programs, and Models of Care to Support Cancer Survivors’ Transition beyond Treatment: A Scoping Review

**DOI:** 10.3390/curroncol28030198

**Published:** 2021-06-09

**Authors:** Claudia Romkey-Sinasac, Stephanie Saunders, Jacqueline Galica

**Affiliations:** 1 School of Nursing, Faculty of Health Sciences, Queen’s University, Kingston, ON K7L 3N6, Canada; sms21@queensu.ca (S.S.); jacqueline.galica@queensu.ca (J.G.); 2 Division of Cancer Care and Epidemiology, Queen’s Cancer Research Institute, Kingston, ON K7L 3N6, Canada

**Keywords:** cancer survivorship, models of care, survivorship care, cancer guidelines, supportive care

## Abstract

(1) Background: One in two Canadians will be diagnosed with cancer in their lifetime, but as a result of the progress in diagnosis and treatment, more individuals are surviving cancer than ever before. However, the impact of cancer does not end with treatment. The objectives of this review are to (1) provide a broad overview of the supportive care interventions and models of care that have been researched to support Canadian post-treatment cancer survivors; and (2) analyze how these supportive care interventions and/or care models align with the practice recommendations put forth by Cancer Care Ontario (CCO) and the Canadian Association of Psychosocial Oncology/Canadian Partnership Against Cancer (CAPO/CPAC). (2) Methods: An electronic search was completed in MEDLINE, Embase, PsycINFO, and CINAHL in January 2021. Included studies described supportive care interventions or models of care utilized by adult Canadian cancer survivors. (3) Results: Forty-two articles were included. Survivors utilized a multitude of supportive care interventions, with peer support and physical activity programs being most frequently cited. Four models of follow-up care were identified: primary care, oncology care, shared-care, and transition clinics. The supportive care interventions and models of care variably aligned with the recommendations set by CCO and CAPO/CPAC. The most commonly followed recommendation was the promotion of self-management and quality resources for patients. (4) Conclusions: Results indicate an inconsistency in access to supportive care interventions and the delivery of survivorship care for cancer survivors across Canada. Current efforts are being made to implement the recommendations by CCO and CAPO/CPAC; however, provision of these guidelines remains varied.

## 1. Introduction

Currently, it is projected that one in two Canadians will be diagnosed with cancer in their lifetime [1]. Fortunately, as a result of progress in diagnostic and treatment options, the incidence of cancer mortality is decreasing, and more individuals are surviving cancer than ever before [1]. The impact that cancer has on one’s life, however, does not end with cancer treatment; many survivors still report challenges and concerns related to their disease as they transition from the end of treatment and into survivorship [2,3]. Cancer survivors indicate that the first year following the end of treatment is difficult [3], as they struggle to adjust to their new normal and experience both physical and psychosocial challenges [3]. As a consequence of this, many cancer survivors rely on their health care provider (HCP) as a crucial source of support throughout their cancer journey [3].

With the increasing incidence and survival rates of cancer, the demand for cancer survivorship care has increased [4]. However, cancer survivorship was not identified as a distinct phase of cancer care until 2006 when the Institute of Medicine (IOM) published the seminal report titled, from cancer patient to cancer survivor: Lost in transition [5]. This report not only defined cancer survivorship care, but highlighted the existing gaps in, and emphasized the need for, a comprehensive, evidence-based approach to survivorship care [5]. The IOM report also outlined common physical and psychological concerns of cancer survivors, the most notable being coping with late and long-term treatment side effects and fear of cancer recurrence [5]. Additionally, it described an optimal approach to cancer survivorship care, which included four components: (1) prevention of new and recurring cancer; (2) cancer surveillance; (3) interventions for cancer and treatment related side-effects; and (4) coordination of care between cancer care and primary care [5]. This report led to an increase in research concerning the survivorship phase of the cancer continuum, and the creation of guidelines outlining the ideal components in the provision of survivorship care.

In 2011, the Canadian Association of Psychosocial Oncology (CAPO) and the Canadian Partnership Against Cancer (CPAC) recognized the need for organized cancer survivorship care in Canada and published the Pan-Canadian Guideline on Organization and Structure of Survivorship Services and Psychosocial-Supportive Care Practices for Adult Cancer Survivors [6]. This guideline was informed by a systematic review which aimed to determine the optimum organization, delivery structure, clinical practices, and interventions to improve or maximize the health and well-being of adult cancer survivors [6]. The guideline proposed several recommendations for both psychosocial and supportive care interventions, as well as recommendations for the organization and delivery of survivorship care, including the integration of primary care providers into the provision of care [6]. This is consistent with findings from Cancer Care Ontario (CCO) [7], which also determined that the traditional specialist/oncologist-led model of follow-up care would become unsustainable with the current landscape of the Canadian health care system [7]. As such, in 2011 CCO supported all fourteen of Ontario’s regional cancer centres in transitioning survivorship care from the traditional oncologist-led model of care, to one of community-based primary care [7]. An analysis into this new model of care revealed that primary care-based survivorship care was safe and effective [7], and was associated with more appropriate use of health care services [8], lower health care costs per survivor [7], as well as a higher rate of appropriate cancer surveillance tests [7,8]. Accordingly, CCO created a set of recommendations to support primary care providers in ensuring that survivors have access to well-informed and high-quality follow-up care [4]. These recommendations by CCO were broadly organized into four categories: follow-up care planning, surveillance, management of consequences of cancer, and health promotion and prevention [4]. 

Even with these evidence-based guidelines from CCO and CAPO/CPAC, which possess many commonalities in their recommendations [4,6], there is variation in the delivery of survivorship care in Canada. Unfortunately, approximately one third of cancer survivors reported that they did not receive the supportive care needed to address their post-treatment concerns [3] and received little guidance from their HCP [9]. This is concerning as cancer survivors are at risk for several physical and psychosocial effects that are unique to their type and stage of cancer, as well as their treatment modality [10]. 

The aim of this scoping review is to provide a description of the current supportive care interventions and transitional care models studied in the context of Canadian cancer survivors and determine how they align with current Canadian guidelines. This will thus illustrate the current landscape of evidence-based post-treatment cancer survivorship care in Canada. Thus, the objectives of this review are (1) to provide a broad overview of the supportive care interventions and models of transitional care that have been researched to support Canadian post-treatment cancer survivors; and (2) to analyze how these supportive care interventions and/or transitional care models align with the practice recommendations by CCO and CAPO/CPAC. The answers to these objectives will illuminate clinical and research areas needed for future post-treatment survivorship care in Canada, as to date, there are no such reviews describing the provision of evidence-based survivorship care in the Canadian context.

## 2. Materials and Methods

### 2.1. Protocol and Registration

The Joanna Briggs Institute scoping review framework was utilized as the guiding methodology to provide a broad overview on what is known about the programs, resources, and models of care to support post-treatment cancer survivors in Canada [11]. To enhance the rigor of this review, the PRIMSA-ScR guidelines for reporting were followed [12], and this protocol was registered publicly on the Open Science Framework (osf.io/aqhxn/, access on 18 August 2020).

### 2.2. Inclusion & Exclusion Criteria

The population of interest for this review was adult (≥18-years-old) cancer survivors who had completed cancer treatment. The concepts of interest were the supportive care interventions (e.g., group exercise classes, educational programs, survivorship care plans, etc.) and/or models of care (e.g., primary care, oncology-based care, shared-care, etc.) that are utilized by cancer survivors as they transition into survivorship care. In identifying these concepts, methods of how the physical and psychosocial supportive care needs of cancer survivors are currently being met in Canada were described. Additionally, this review only included Canadian articles, so as to provide an overview of current cancer survivorship care within the Canadian context specifically. Included material was limited to full text, English language, primary research articles. There was no limit placed on the date of publication to provide a wide image of survivorship care in Canada. This review excluded studies where post-treatment data were not clearly extractable, such as in studies that included both individuals undergoing treatment and those who had completed treatment.

### 2.3. Search Strategy

A literature search was completed in MEDLINE, Embase, and PsycINFO databases on the Ovid platform, as well as the Cumulative Index of Nursing and Allied Health Literature (CINAHL) on the EBSCOhost platform. Search strategies were adapted to meet the needs of each individual database. The search was completed in June 2020 and updated in January 2021. The search strategy utilized in Ovid MEDLINE is displayed in Table S1. 

### 2.4. Study Selection & Data Extraction

Following the search, the identified research articles were imported into the reviewing software, Covidence [13]. Two reviewers, C.R.S. and J.G., both independently screened all titles and abstracts, followed by the full text, to determine if the studies met the inclusion and exclusion criteria. The same two reviewers (C.R.S. and J.G.) then independently extracted data from the eligible studies, using a custom Excel form that had been previously pilot tested by the reviewers. This form included: (1) lead author, (2) year of publication, (3) province of origin, (4) research methodology, (5) aims/purpose, (6) study population (age, patient/partner, type of cancer diagnosis, and sample size), and (7) key findings that related to the review objective (see Table S2). All conflicts throughout the process were resolved through consultation with the third reviewer (S.S.) to improve interrater reliability.

### 2.5. Data Analysis

Each included article was categorized based on whether the extracted content was describing a supportive care intervention or transitional model of care. Thereafter, a thematic grouping of articles within each categorization was conducted to address the first objective of the review.

The CCO [4] and CAPO/CPAC guidelines [6] were reviewed and the recommendations grouped by similarities. This resulted in a total of 26 unique recommendations for the provision and organization of cancer survivorship care. This process is available in Table S3. Recommendations at the policy or systems level were omitted (e.g., promoting awareness of survivorship issues, evaluation of survivorship services, etc. [6]). Authors (C.R.S. and J.G.) then mapped the content of the included articles to each of the 26 recommendations, indicating whether the recommendation was met, partially met, or not at all described within the content of the article. Independent confirmation was then conducted by the third reviewer (S.S.). This provided a descriptive analysis of which CCO [4] and CAPO/CPAC [6] recommendations were being met or did not describe being met, thus addressing the second objective of the review. 

## 3. Results

**Figure 1:**
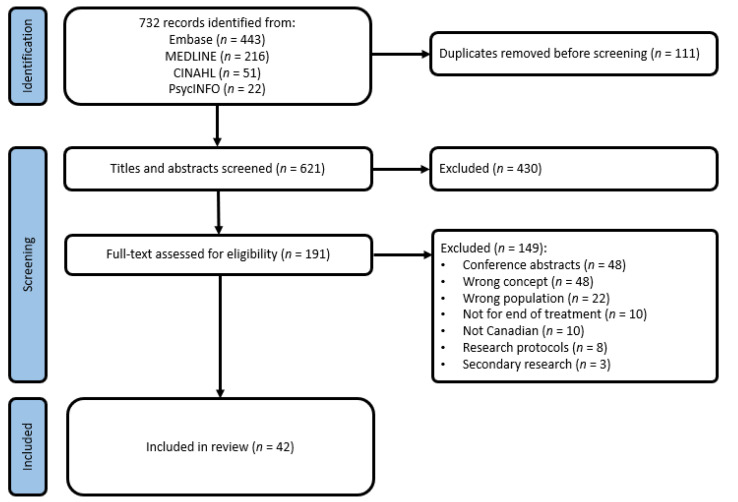
PRISMA Flow Diagram.

A total of 732 articles were identified as eligible for screening (Figure 1). After the removal of 111 duplicates, 621 studies remained and advanced to title and abstract screening. Four-hundred and thirty studies were deemed irrelevant and 191 full text articles were assessed for eligibility. A total of 42 articles were included in this review (see Table S2). Reasons for exclusion are reported in the PRISMA Flow Diagram (Figure 1). 

Of the included articles, 22 (52%) were published within the last five years (2016 or later), and the remaining papers (*n* = 20) were published since 2003. Most (*n* = 20, 48%) studies used a quantitative design (e.g., four RCTs, two single-arm interventions, five administrative data or chart reviews), 15 (36%) employed qualitative methods, and mixed or multiple methods were utilized in the remaining seven studies (17%). Most studies were completed in Ontario (*n* = 13, 31%) or Alberta (*n* = 9, 21%), and another eight (19%) were pan-Canadian or involved multiple provinces. None of the studies explicitly reported on populations from Saskatchewan or the Territories. The most commonly studied cancer survivor population was breast cancer (*n* = 17, 41%), followed by multiple cancers (*n* = 11, 26%). One study did not report on the type of cancer. 

### 3.1. Models of Care

The reviewed papers revealed four models of care utilized in the provision of cancer survivorship care in Canada: discharge direct to primary care (*n* = 6 [14,15,16,17,18,19]); follow-up care facilitated through a transition clinic at the cancer centre (*n* = 5 [15,20,21,22,23]); remaining in oncology-led care (*n* = 3 [14,19,24]); and shared-care between oncology and primary care (*n* = 3 [14,15,19]). Direct to primary care was the most commonly discussed model of care [14,15,16,17,18,19] wherein survivors were discharged from their oncologist directly into the care of their primary care provider (PCP) (i.e., general practitioner or nurse practitioner) with no routine follow-up from oncology [15]. Remaining in the care of the oncologist was the preferred model of care for many cancer survivors [14,25]. Ontario had the highest proportion of survivors who solely saw an oncologist annually for follow-up care [24]. A shared-care model was also commonly presented wherein the goal was to gradually transition care from the oncology team to PCP within five years after the end of treatment [15]. In some regions (e.g., Ontario [15,22,23] and Manitoba [21]), the shared-care model was facilitated by a transition clinic at the cancer centre [15], which was intended to be a transitional service between oncology and primary care [15,21,22,23]. The intent of the transition clinic was to enhance the collaboration and communication among the survivor, cancer centre clinicians, and the PCP [15,21]. In this model they were provided with individualized information about cancer survivorship [21], resources [21], and a patient specific survivorship care plan (SCP) [21,26]. Additionally, survivors were offered a place to ask questions about their follow-up cancer care [21] and receive individualized supportive care interventions [21]. 

### 3.2. Supportive Care Interventions

To meet their need for supportive resources, results revealed that cancer survivors used physical activity programs (*n* = 11, 26%) [27,28,29,30,31,32,33,34], such as dragon boat racing [27], group-based exercise classes [32], brisk walking [29,31], yoga [31], and resistance training [30,31]. Three other articles described educational programs for cancer survivors related to fatigue management [35], sexual health [36], and relevant matters to cancer survivors [37]. A further article outlined a joint educational and physical activity program [35]. However, many survivors claimed that they were not provided with information about, or were unaware of, programs or resources that could be useful to support them beyond the end of treatment [38,39,40,41,42], which left them feeling as though they were responsible for finding their own support [38,39]. Rural [25,39] and Indigenous [40] cancer survivors reported additional challenges in accessing resources and programs for their follow-up care needs. These challenges included increased transportation [39,40] and financial burdens [39,40], and difficulty in accessing HCPs themselves [25,39,40], as these services were not available locally in their communities [25,39,40]. In addition to organized programs, cancer survivors accessed support from their own personal resources. They received social [43], emotional [43], and tangible support [43,44] from family members and spouses [17,38,43], friends [43,44,45], other cancer survivors [25,38,43,44,46], support groups [25,44,46], and through volunteer work [25,44]. Spirituality, faith, and religion were discussed in several articles as a resource to help survivors cope with and find meaning in their illness [17,38,47,48,49]. This included activities such as attending a place of worship [47,49], praying [47,49], singing [47,49], and practicing mindfulness [38]. 

Some survivors sought counselling from their HCPs [17,43,46,50] for issues such as emotional distress [46] and lifestyle modification (e.g., smoking cessation) [50]. Other survivors sought information from print and electronic sources, such as research articles [25], pamphlets [25,51], books [25,38], the Internet [25,38,45,51,52], and teleconferences [25], which gave them confidence to make informed decisions about their care [25]. SCPs were a resource [17,51,52,53,54,55] that some survivors cited as useful in their transition to follow-up care [17,53]. 

### 3.3. Alignment with CCO & CAPO/CPAC Recommendations

The supportive care interventions and models of care outlined in the reviewed papers variably aligned with the elements of current Canadian cancer survivorship recommendations (Table S4) [4,6]. The most common recommendation addressed in the reviewed papers was the provision of self-management and quality resources for patients (CCO-15, A-6, B-1). This was addressed fully or partially in 16 (38%) of the included studies [15,21,22,23,26,29,30,32,34,36,37,46,49,51,53,56]. Three recommendations were equally addressed in the reviewed papers: ‘to support healthy behaviours (B-1)’ [17,23,28,29,30,34,37,50,52,53]; to provide ‘variable delivery of supportive care and information (CCO-14)’ [14,22,27,37,38,45,46,52,56,57]; and ‘treatment summary, follow-up plan and contacts given to patient (CCO-4, A-3)’ [15,17,18,21,22,26,51,52,53,55]. These recommendations were each reflected in 10 (24%) of the included papers (Table S4). Notably, three recommendations were not reflected in any of the reviewed papers: appropriate sharing of surveillance test results with patients (CCO-11); managing vasomotor symptoms (B-7); and programs based on behaviour change theories (B-2). 

## 4. Discussion

This scoping review provides a description of the current supportive care interventions and transitional care models studied in the context of Canadian cancer survivors. It presents an overview of how these interventions align with current Canadian guidelines, thus illustrating the current landscape of evidence-based post-treatment cancer survivorship care in Canada. However, current follow-up care recommendations by CCO and CAPO/CPAC have been met to varying degrees, and implementation is inconsistent from one region to the next. By describing the current landscape of survivorship care, we have identified current gaps and strengths in the provision of cancer survivorship care in Canada, as well as areas for future research. 

Within this review four models of care were researched in Canada. However, survivors reported a preference for receiving follow-up care from their oncologist at the cancer centre [14,25], as they perceived the oncologist as having more expertise in cancer care and knowledge about their individual case [14]. Nevertheless, a systematic review [7] reported no difference in cancer survivor quality of life nor practitioner’s ability to detect cancer recurrence among those who received primary care as opposed to oncologist follow-ups. This indicates the suitability of PCPs to provide survivorship care, and qualifies them as a viable option to reduce the strain on the traditional oncologist-led model of care [14]. Indeed, such a transition in care is supported by the CCO and CAPO/CPAC recommendations examined in this review, pointing to the appropriateness of a shared-care or transitional model of care from oncologist to PCP care. This may aid in reducing the hesitation survivors experience when returning to primary care [14]. These models are demonstrably feasible and achievable in the Canadian health care system [15], and are associated with a decrease in distress experienced by cancer survivors in the follow-up period [23]. 

While not frequently discussed in the articles included in this review, telehealth is becoming a common care delivery method in the wake of the COVID-19 pandemic. Conversion to telehealth has been shown to increase participation in programs, as this delivery method makes them accessible from home [58], and reduces barriers such as transportation costs and time burdens [58]. However, some survivors reported that they preferred in-person services [14,58], as they feared the loss of the physical exam portion of the visit [14]. It is important to remember that each survivor is unique, and that their experiences and needs differ, as do their preferred care delivery modalities. Regardless of the model of care, survivors should be aware of which HCP is most responsible for their follow-up care, while maintaining an open line of communications among the oncologist, PCP, and survivor.

This review identifies a number of supportive care interventions utilized by cancer survivors. However, cancer survivors report being largely unaware of resources or programs that are available to them [38,39,40,41,42]. Some papers alluded to survivors’ self-initiated actions to engage in activities that aligned with CCO or CAPO/CPAC recommendations, which these organizations claim that professionals should promote. For instance, CAPO/CPAC recommendation A-6 [6] indicates that providers should focus on ’enabling and empowering individuals’ to be active in optimizing their health and wellbeing, when in fact some survivors may already be doing this without the involvement of their HCPs. This may indicate that the recommendations put forth by CCO and/or CAPO/CPAC need to be adapted to speak to survivors who are self-motivated to engage in their self-management. Understanding the traits of such survivors would also be an important area of further study.

Although most CCO [4] and CAPO/CPAC [6] guidelines are reflected in the reviewed studies, some recommendations are not or are poorly represented (e.g., sharing of surveillance test results with patient, or managing vasomotor symptoms). Notably, these recommendations may be addressed by providing such information in a living document or plan of the ongoing care needs of the cancer survivor that is updated as their needs change. Indeed, CCO [4] and CAPO/CPAC [6] recommend that a SCP be implemented at the end of active treatment in order to create an open channel of communication between the survivor and practitioners. Although these guidelines identify the type of information that should be included in the SCP (e.g., cancer diagnosis and treatment received, recommended follow-up timelines, etc. [5]), the results of this review suggest that SCP content may need modifications to include a greater number of CCO and CAPO/CPAC recommendations (e.g., the recommendations not identified in the included studies). Nevertheless, implementation of SCPs is neither standard nor consistent across Canada [41], likely because there is limited evidence to substantiate their use [59]. Regardless, cancer survivors have identified SCPs as something they wish to have implemented in the future [38,41], which may be particularly important in providing standardized but individualized care regardless of geographical location across the country. 

This review highlights that survivors living in rural settings do not have the same access to care as their metropolitan counterparts [25,28]. Indeed, rural Canadian cancer survivors face increased transportation and financial challenges in accessing follow-up and rehabilitative care, as resources or specialists are not available locally and they had to travel to larger communities to receive care [25,39,40]. Survivors in remote communities reported that HCPs would fly into the community for a follow-up visit; however, each time there would be a different provider, leaving survivors to explain their medical history at each visit [25]. This lack of continuity led to increased anxiety for survivors and a feeling as though they were not receiving the same standard of care as their urban counterparts [25]. These issues were also reported to be the case in the context of Indigenous cancer survivors [40]. Further issues experienced by Indigenous survivors included a lack of culturally competent care which perpetuated their anxiety in accessing care [40]. To mitigate the negative consequences of these geographical disparities, and to promote the self-management and resourcefulness of cancer survivors in a diversity of settings, further examination of the SCP is warranted.

### 4.1. Implications for Practice and Research

With one in two individuals expected to be diagnosed with and survive cancer in their lifetime [1], it is imperative that the provision of survivorship care is delivered in a sustainable and evidence-based manner. This is especially true for publicly funded health care systems—such as in Canada—wherein the population is intended to have reasonable access to care without paying for services. Thus, with increased numbers of cancer diagnoses and survivors, oncology-based follow-up care has largely been slated as unsustainable [5], leading to a push for primary care-based survivorship care. Evidence-based recommendations have been set forth by organizations such as CCO [4] and CAPO/CPAC [6] to aid in this transition from oncology to primary care. However, through this review it was identified that the content of three recommendations by CAPO/CPAC [6] and CCO [4] were not discussed/met in any of the included manuscripts (Table S4), indicating that these recommendations reflect current gaps in the provision of survivorship care in Canada.

The first of these was recommendation B-2, which promotes the use of health behaviour change theories to influence the adoption of healthy lifestyle behaviours in cancer survivors [6]. It is notable that no program within this review reported using a health behaviour change theory in their methodology. A systematic review by Pinto and Floyd [60] showed that when behaviour change theories are used as a guiding framework in the development of lifestyle interventions, cancer survivors are more successful in improving their fitness, maintaining a healthy diet, achieving an ideal body weight, reducing pain and fatigue, and improving overall health and vigour. Additionally, a review by Graves et al. [61] determined that psychosocial interventions based on social cognitive theory have a greater effect on reducing depression, as well as improving social, physical, and quality of life outcomes in individuals with cancer. These findings indicate that health behaviour change theories provide a knowledge base and framework that has great utility in increasing uptake and efficacy of psychosocial and lifestyle interventions in the cancer population.

The appropriate sharing of surveillance test results with the survivor (CCO-11) [4] was another recommendation not identified in the results of this review. Some survivors reported that surveillance tests create increased anxiety relating to the fear of cancer recurrence [62], which is reportedly a contributing factor to the poor uptake of surveillance testing in the cancer survivor population [63]. A positive relationship [62] and effective communication [63] between survivors and their HCPs was reported to help mitigate the survivor’s fears and anxiety related to surveillance testing [63], ultimately increasing their self-efficacy in managing their health [64]. It is therefore important for HCPs to understand that surveillance testing is an anxiety-provoking event and to appropriately share surveillance results with survivors in a timely manner so as to lessen their fears and improve their self-management. 

The last recommendation that was not addressed in the literature of this review was the management of vasomotor symptoms. The CAPO/CPAC recommends that all female cancer survivors have access to cognitive behavioural therapy and lifestyle management programs targeted in alleviating vasomotor symptoms [6]. However, this recommendation is based solely on findings specific to breast cancer survivors [6]. This highlights a limitation present in this review: the disproportionate representation of breast cancer survivors. This consequently reduces the generalizability of this recommendation to cancer survivors of all types. 

### 4.2. Limitations

This scoping review is the first to highlight the landscape of survivorship care in the Canadian context, which has important research and practice implications. However, this review is not without limitations. Due to the scoping review methodology, a critical appraisal of the literature was not completed, providing no indication of the quality of the included studies describing the programs, resources, and models of care. However, this was not the intent of the current study. Instead, the authors strove to be inclusive of all peer-reviewed studies conducted in this realm across Canada so as to illuminate the areas researched and in need of further research.

While this review included all types of cancers so as to keep the picture of survivorship care in Canada broad, the majority of available Canadian literature focuses on breast cancer survivorship specifically. Forty-one percent of articles included in this review contained breast cancer survivors as their population of interest, and a large proportion of the evidence informing this review is biased towards breast cancer survivorship as discussed above. This limits the generalizability of this review to other cancer types, as it overrepresents the needs and experiences of breast cancer survivors. Similarly, many of the studies were conducted in large urban centres in Ontario, and only a small proportion discussed care of rural cancer survivors; this overrepresents the urban cancer survivor experience. Moreover, the Territories and Saskatchewan were not represented within the review, and only two articles [40,47] reported on the experiences of Indigenous populations. Given that Indigenous peoples and individuals living in rural and remote areas of Canada have different experiences interacting with and accessing the health care system than individuals living in large urban areas [25,39,40], there is a need to further explore and include these population in future cancer survivorship research. 

Additionally, the scope of information available is limited to the content presented in the reviewed articles. Therefore, some of the recommendations by CCO and the CAPO/CPAC may have been adhered to but not reported by authors, and as such not included in the analysis of this review. Furthermore, although population database examinations were useful to underline the supportive care interventions and models of care used by cancer survivors, these types of studies do not permit the examination of the applicability of the guidelines.

## 5. Conclusions

With the projected increase in the number of individuals surviving cancer [1], the traditional model of oncologist-led survivorship care has been largely viewed as unsustainable in Canada [5], creating an ongoing need to invest in survivorship care [1,3]. This review aimed to provide a descriptive summary of the current supportive care interventions and transitional care models utilized by cancer survivors in Canada, and illustrate how they align with current practice guidelines. The findings of this review may help in the future coordination of follow-up cancer survivorship care in Canada and to identify the current strengths and gaps in research and practice, including an inconsistency in the delivery of survivorship care throughout geographical regions within Canada. In doing so, cancer survivors in Canada will receive comprehensive, evidence-based care as they transition from the end of treatment and into survivorship [1,4,5,6].

## Data Availability

Not applicable.

## Supplementary Materials

The following are available online at https://www.mdpi.com/article/10.3390/curroncol28030198/s1. Table S1. MEDLINE on Ovid search strategy, Table S2. Synopsis table of included studies. Table S3. Process for coding and combining cancer survivorship recommendations by CCO [4] and CAPO/CPAC [6]. Table S4. A mapping of cancer follow-up recommendations [4,6] reflected in the reviewed papers.
