# Supplementary material for: Canadian Resources, Programs, and Models of Care to Support Cancer Survivors’ Transition beyond Treatment: A Scoping Review
Source: Curr Oncol. 2021 Jun 9;28(3):2134–45. doi: 10.3390/curroncol28030198 (PMC8293069; doi:10.3390/curroncol28030198)
Supplement: Supplementary file 1 [file curroncol-28-00198-s001.zip › curroncol-1226391-supplementary.pdf]

Current Oncology

MDPI

Review

Canadian Resources, Programs, and Models of Care to Support Cancer Survivors' Transition beyond Treatment: A Scoping Review

Claudia Romkey-Sinasac, Stephanie Saunders and Jacqueline Galica

Supplementary Materials

Table S1. MEDLINE on Ovid search strategy.

Search ID	Search Strategy
S1	((cancer.mp. or exp Neoplasms/AND survivor.mp. or exp Survivors/) OR (cancer survivor.mp. or exp *Cancer Survivors/) OR (cancer survivor.mp. or exp * Cancer Survivors/)) OR (caregiver.mp. or exp Caregivers/or support person.mp. or partner.mp. or spouse.mp. or exp Spouses/)
S2	exp Primary Health Care/or health care services.mp. or health care delivery.mp. or exp "Delivery of Health Care"/or model of care.mp.
S3	mental health services.mp. or exp Mental Health Services/or Community services.mp. exp Community Health Services/or Community programs.mp. or Cancer program.mp. or support group.mp. or exp Self-Help Groups/
S4	(health resources.mp. or exp Health Resources/or needs assessment.mp. or exp Needs assessment/or survivorship care plan.mp. or care pathway.mp. or clinical practice guideline.mp. or exp Practice Guideline/) OR ((supportive care.mp. or support.mp. or unmet.mp. or information.mp. or communication.mp. or exp Health Communication/or exp Communication/or emotional.mp. or psychological.mp. or psychosocial.mp. or exp Psychosocial Support Systems/or psychosexual.mp. or exp Sexual Health/or sexual*.mp. or relationship.mp. or spiritual.mp. or exp Spirituality/or existential.mp. or cultural.mp or physical.mp. or exp "Activities of Daily Living"/or daily living.mp. or functional.mp. or quality of life.mp. or exp "Quality of Life"/or exp Health Promotion/or wellness.mp. or well being.mp. or weight.mp. Or exp Exercise/or exercise.mp. or activity.mp. or diet.mp. or exp Diet/or smoking.mp. or exp Smoking/or alcohol.mp. or self care.mp. or exp Self Care/or symptom management.mp. or side effect*.mp. or body image.mp. or exp Body Image/or fertility.mp. or exp Fertility/or fear of recurrence.mp. or fear of cancer recurrence.mp.) AND (intervention.mp. or need*.mp. or support.mp.))
S5	survivor* care.mp. or cancer surveillance.mp. or transition to survivor*.mp. or cancer survival.mp. or care transition.mp. or exp Patient Transfer/or transitional care.mp. or exp Transitional Care/or care transfer.mp. or following treatment.mp. or post treatment.mp. or after treatment.mp. or exp Aftercare/or follow* care.mp.
S6	exp Canada/or Canada.mp or Canadian.mp
S7	S1 AND (S2 OR S3 OR S4) AND S5 AND S6
S8	Limit S7 to (English language and "all adult (19 plus years)") (204)

Table S2. Synopsis table of included studies.

Author Year Province	Purpose/Objective	Sample Size and Characteristics	Results Relating to Study Objectives
Alimujiang [49] 2019<br><br>Alberta	The purpose of this study was to gather information on the lifestyle, behaviors, and personal care factors that women think influenced their exceptional survival with ovarian cancer.	*n* = 26<br>Ovarian (100%)<br>Age (mean 52)<br>Yr since dx:<br>5–7 yr = 5,<br>8–9 yr = 2,<br>10 yr or more = 19	- Survivors discussed changes that they made to their lifestyle after cancer including diet, exercise, and supplements.<br>- Family, friends, and HCPs became more important as a source of support.<br>- Church was important for a source of spirituality, and social support.

Bender [14]<br>2016<br><br>Ontario	The purpose of this study was to examine WDTC survivors' perceived satisfaction with follow-up care involving different clinicians and mediated by the Internet, and their perceptions of follow-up care options.	*n* = 202<br>Thyroid (100%)<br>Age (mean 50.1, SD 14.7)<br>Yr since dx (median 3.6, range 3 mo–57 yr, IQR = 4.9)	- Discussed what interactions cancer survivors had with HCPs in the last year.<br>- Survivors preferred to receive follow-up care in-person, and with their specialist.
Bilodeau [42]<br>2019<br><br>Quebec	The goal of the present article was to outline gaps and delay in survivorship care in the return to work pathway for survivors of breast cancer.	*n* = 9<br>Breast (100%)<br>Age (mean 49 yr, range = 30–60)	- Many survivors experience late and long-term side effects of their cancer tx.<br>- Survivors discussed the lack of information or support locating resources at EOT.<br>- Discussed wanting a program or a resource person to connect them to resources.<br>- Survivors reported not receiving an SCP.
Boekhout [55]<br>2015<br><br>Western Canada, Ontario, Quebec, Atlantic Canada	This study aimed to present the 24 months results of health services and patient-reported outcomes of cancer survivors being transferred from follow-up care to their primary care physicians.	*n* = 337<br>Control (*n* = 173)<br>Breast (100%)<br>Age (mean 61.9 yr, SD 10.2, range 38.1–87.5).<br>Time since dx<br><24 mos: *n* = 69, mean = 11.3 range 3.5–24;<br>>24 mo = 104, mean =79.1 range 3.7–300<br>Intervention (*n* = 164)<br>Breast (100%)<br>Age (mean = 61.1, SD 10.2, range 35.6–87.8).<br>Tme since dx<br><24 mo: *n* = 70, mean= 12.3, range =3.6–29);<br>>24 mo: *n* = 94, mean= 83.4, range = 11.8–375)	- Intervention group received a SCP, which produced no difference in cancer-specific distress, psychological distress, health-related quality of life, patient satisfaction, or continuity and coordination of care.<br>- SCP contained a personalized tx summary, a patient's version of the Canadian follow-up guidelines, and a resource kit.<br>- SCP was given to both the patient and PCP at the EOT, and the patient received an educational session with the oncology nurse.
Bowler [53]<br>2019<br><br>Alberta	This study aimed to identify the satisfaction with and effectiveness of the AHS self-management guidelines for the genitourinary population receiving curative EBRT.	*n* = 18<br>Prostate (100%)<br>Age<br>45–54 yr = 2,<br>61–70 yr = 9,<br>71–74 = 4<br>≥ 75 = 3	- Prostate cancer survivors were provided with the Alberta Health Services self-management guidelines following EOT.<br>- Information in the guideline included: follow-up care information, side effects, genetics, fertility, sexuality, living with hope and uncertainty after tx, resources for support, reducing risk of recurrence, and additional health resources.<br>- Over 90% of survivors found the guidelines useful.
Brunet [32]<br>2016<br><br>Ontario	We conducted a longitudinal qualitative study with the aims of understanding breast cancer survivors' perceptions of the instructor and the climate the instructor created within	*n* = 7<br>Breast (100%)<br>Age (mean 55.3, range 40–69 yr)	- Breast cancer survivors participated in an eight-week group-based exercise program at the Ottawa Regional Cancer Foundation.<br>- Survivors reported that the instructor led them to achieve their own individual exercise goals even while in a group setting.

	the context of a group-based exercise program and how this contributes to women's motivational experiences.		
Chahine [54] 2019<br><br>Nova Scotia	The objective of this study was to evaluate the impact of receiving an SCP on meeting the overall, informational, physical, emotional, and practical needs of survivors' post-treatment.	*n*= 1514<br>Cancer Type:<br>Breast (*n* = 473, 31.2%)<br>Colorectal (*n* = 332, 21.9%)<br>Melanoma (*n* = 148, 9.8%)<br>Prostate (*n* = 384, 25.4%)<br>Other (*n* = 176, 11.6%)<br>Age:<br><34yr = 23 (1.5%)<br>35–64yr = 499 (33.2)<br>65–84yr = 911(60.6%)<br>>85yr = 70 (4.7%).<br>Time since tx<br><1 yr = 141 (9.6%)<br>1–2 ys = 208 (14.2%)<br>2–3 yr = 455 (31.1%)<br>>3 yr = 354 (24.2%)<br>No tx= 305 (20.9%)	- Study examined the effects of receiving an SCP.<br>- Almost 90% of participants expressed that they received fu care that met their needs.<br>- Participants who received an SCP were more likely to have their overall needs met than individuals who did not receive an SCP.<br>- Younger patients were more likely to not have their needs met in the post-tx stage.
Chongya [50] 2015<br><br>Ontario	The study aim was to determine whether differences existed between younger and older patients with cancer with respect to: smoking cessation, alcohol moderation, and regular physical activity; their knowledge of and attitudes toward these behavioral changes; and the frequency of receiving any information or counseling on these behaviors from their health care providers after their cancer diagnosis.	*n* = 616<br>Cancer Type:<br>Breast (15%)<br>GI (14%)<br>Genitourinary (13%)<br>Gynecologic (6%)<br>Head and neck (9%)<br>Hematologic (24%)<br>Lung (4%)<br>Skin and sarcoma (8%)<br>Thyroid (6%)<br>Age<br>younger pt <65 yr *n*= 477<br>elderly pts >65 yr *n* = 139	- Study compared the difference between younger (<65) and older (≥65) cancer survivors and their health-related behaviors pertaining to smoking, alcohol consumption, and physical activity.<br>- Almost half of both older and younger cancer survivors quit smoking after dx, and many received counselling from their HCP in order to do so.<br>- Older survivors were less likely to modify their physical activity levels to meet suggested activity guidelines.
Collie (51) 2014<br><br>Alberta	The purpose of the qualitative component was to hear directly from survivors, nurses, and family physicians about their experience with the content and delivery of the SCPs.	Questionnaires (*n* = 54)<br>Interviews (*n* = 12)<br>Head and neck (*n* = 4, 33%)<br>Breast (*n* = 8, 67%)<br>Breast Age (mean = 53.6 yr ± 9.29.	- Created an SCP based on the CAPO/CPAC and IOM recommendations, and included:<br>1. dx info and tx info.<br>2. Guidelines for fu care.<br>3. Care team contact info.<br>4. Coping and adjustment recommendations.<br>5. Healthy living recommendations.<br>6. Resources.<br>- Survivors reported being satisfied with the SCP they received.

		Head and neck Age (mean = 55.5 yr ± 11.90)	
Cyr [56] 2020<br><br>Quebec	To develop a multimodal pelvic floor physical therapy intervention and to examine its feasibility and acceptability in gynecological cancer survivors with dyspareunia as well as its effects on pain, sexual function, pelvic floor dysfunction symptoms, and related impact on quality of life.	*n* = 31<br>Endometrial *n* = 20 (64.5)<br>Cervical *n*= 11 (35.5)<br>Age = 55.9 ± 10.8<br>Time since tx (38 mo)	- Participants with dyspareunia completed a 12-week pelvic floor physical therapy intervention, which included education, manual therapy, pelvic floor muscle exercises using biofeedback, and home exercises.<br>- There was significant reduction in pain quality and intensity.<br>- Sexual functioning improved and there was an increase in frequency of sexual activities with vaginal penetration.<br>- Women experienced fewer urinary, vaginal, and bowel symptoms.
Dawe [43] 2014<br><br>Newfoundland	The purpose of this paper is to report the findings of a study about the informational and emotional needs of women having outpatient surgery for breast cancer.	*n* = 19<br>Breast (100%)<br>Age (mean = 57, range 38–72 yr)	- Survivors discussed their emotional and informational needs prior to and immediately after surgery, while recovering at home, and as an outpatient.<br>- The social support network of survivors included spouses, family, HCPs, and other survivors.
Easley [39] 2012<br><br>Atlantic Canada (PEI, NS, NB, NL)	The purpose of this study was to assess the rehabilitation needs and preferences of young women under the age of 50 with breast cancer in Atlantic Canada and to identify factors that may impact or prevent cancer rehabilitation utilization.	*n* = 35<br>Breast (100%)<br>Age (mean = 42 yr, SD 6.05)	- Survivors discussed issues with rehabilitative care following breast cancer including lack of awareness of services, financial challenges, lack of continuity of care and communication among HCPs and with patients, and limited access in rural areas.<br>- The top 5 desired rehabilitation services survivors believed should be included in cancer care were:<br>1. Physiotherapy<br>2. Psychologist<br>3. Sexuality/Fertility Counseling<br>4. Exercise Program/Personal Trainer<br>5. Massage Therapy/Manual Lymph Drainage
Farris [33] 2017<br><br>Alberta	We examined the role of both total post-diagnosis physical activity and each type of activity including recreational, occupational, and household activity, and changes in physical activity over the diagnostic period on physical and mental QoL in prostate cancer survivors.	*n* = 817<br>Prostate (100%)<br>Age (mean = 67.3 yr)	- Investigated the impact of physical activity on a number of domains (psychological, physical, etc.).<br>- Recreational activity was found to increase both physical and mental functioning.<br>- Those who adhered to the physical activity guidelines obtained higher scores in physical functioning and quality of life than non-exercisers.
Fillion [35] 2008<br><br>Quebec	The purpose of this randomized control trial was to verify the effectiveness of a brief group intervention that combines stress management psycho-education and physical	*n* = 87<br>Breast (100%)<br><br>Experimental Group *n* = 44<br>Age (mean = 53.09 yr, SD 9.65)	- Examined the effects of a four-week group intervention that combined stress management, psycho-education, and physical activity.<br>- The intervention was composed of 4 weekly group meetings of 2.5 hours and 1 short telephone booster session. One hour was devoted to the supervision of walking training by a kinesiologist or a trained research nurse, and 1.5 hours to the psycho-educative,

	activity (ie, independent variable) intervention in reducing fatigue and improving energy level, quality of life (mental and physical), fitness (VO2submax), and emotional distress (ie, dependent variables) in breast cancer survivors.	Control Group *n* = 43<br>Age (mean = 51.84 yr, SD 10.25)	fatigue management sessions, which were codirected by 2 oncology nurses.<br>- Women who received the intervention showed increased physical quality of life and higher energy levels, as well as decreased fatigue and emotional distress.
Galica [38]<br>2020<br><br>Ontario	The objective of this qualitative descriptive study was to explore how ovarian cancer survivors living in smaller urban or rural areas cope with fear of cancer recurrence.	*n* = 15<br>Ovarian (100%)<br>Age (mean 62.8 yr, SD 6.6, range 51–76 yr)<br>Time since dx (mean= 2.7 yr, SD 4.6, range 1–19)	- Survivors relied on their HCPs as a means of support, education, and reassurance.<br>- They identified literature freely available in clinic areas, websites, books, or workshops at the cancer centre as a means of information and coping with their fear of cancer recurrence.<br>- Women discussed finding emotional support from their family and other cancer survivors.<br>- Women discussed wishing they had received an SCP, to help them navigate the transition to survivorship and help prepare for their post-tx expectations.
Gall [48]<br>2004<br><br>Ontario	The present study represented an initial exploration of the potential role of one religious/spiritual factor, religious coping, in men's long-term adjustment to prostate cancer.	*n* = 34<br>Prostate (100%)<br>Age (mean =65.8 yr, range 47–77)	- The use of religious coping behavior was related to lower levels of role, social, and emotional functioning for prostate cancer survivors.<br>- Spirituality-based coping was significantly related to seeing the illness more positively.
Gaudine [25]<br>2003<br><br>Atlantic Canada (PEI, NS, NB,NL)	The purpose of the study was to determine, through women's own voices and descriptions of their experiences, the interplay of the anxiety/reassurance equilibrium that accompanies waiting for general physical and medical tests and clinic visits over an extended period of time.	*n* = 9<br>Breast (100%)<br>Age<br>31–49 yr = 4,<br>50–54 yr = 5<br>Time since dx<br>2–4 yr ago = 7<br>7 yr ago = 2	- Women reported a need for increased support throughout their cancer experience, and reported finding this in their husbands, loved ones, HCPs, and through volunteering and support groups.<br>- Survivors looked for information about their cancer and side effects in research articles, pamphlets, books, the Internet, and teleconferences.<br>- Cancer survivors preferred to receive their fu care at the cancer clinic, as they felt a sense of belonging and maintained continuity of care in this setting.<br>- Survivors recommended that each survivor be given a schedule and information on what to expect in the fu period at the beginning to help lessen anxiety.
Gifford [47]<br>2019<br><br>British Columbia, Ontario, Quebec	The purpose of this study was to explore the meanings of spirituality in cancer survivorship healing with First Nations people, the largest Indigenous population in Canada	*n* = 31<br>Cancer survivors *n* = 29<br>Caregivers *n* = 2<br>Cancer Type:<br>Breast *n* = 19<br>Urinary *n* = 4<br>GI *n* = 3	- Study described the spirituality of First Nations cancer survivors.<br>- Survivors spoke of different practices in which they engaged to incorporate spirituality into cancer survivorship including giving thanks, attending places of spiritual connectedness, singing, praying, and engaging with the sun and moon.

		Respiratory *n* = 2, Leukemia/lymphoma *n* = 2<br>Skin *n* = 1<br>Age (median 59.5, range 27–82)	
Goldenberg [21]<br>2020<br><br>Manitoba	The program evaluation addressed whether receiving a survivorship care plan at the time of discharge from primary care is associated with greater perception of continuity of care compared to standard of care.	*n* = 246<br>Pre-implementation group<br>Colon (*n* = 152, 61.8%)<br>Rectum (*n* = 94, 38.2%)<br><br>*n* = 83<br>Post-implementation group<br>Colon (*n* = 36, 43.4%)<br>Rectum (*n* = 47, 56.6%)	- Followed colorectal cancer survivors through the implementations of a patient-centred transition program, including an SCP.<br>- SCP content included information on diet and nutrition, fu care and side effects, exercise and activity, specific resources available in Manitoba, emotional and psychological impact of cancer and tx, wellness and return to work, navigating primary care, reducing risk of recurrence, sleeping well, and health care decisions and considering the future.<br>- The majority of survivors felt as though they were adequately prepared for having their fu care transferred and were given information on fu appointments, support services, and fu tests.
Grant [15]<br>2015<br><br>Ontario	Described the formative evaluation of an initiative involving the development and implementation of sustainable models of follow-up care for BC survivors across 14 regional cancer centres (RCC) in Ontario, Canada	*n* = 3,138<br>Breast (100%)<br>Age:<br><46 yo = 3%<br>46–55 yo = 16%<br>56–65 yo = 29%<br>66–75 yo = 31%<br>76–85 yo = 17%<br>≥86 = 4%	- Discussed the three possible models of care that survivors experienced during the transition to follow-up care in Ontario: 1) directly to primary care, 2) shared-care, and 3) transition clinic.<br>- All models of care in the study provided survivors with an individualized SCP, transition letter, and educational material.
Hammond [40]<br>2017<br><br>British Columbia, Ontario, Quebec	Explored survivorship experiences of First Nations women with cancer and their caregivers.	*n* = 58<br>*n* = 27 cancer survivors<br>*n* = 31 caregivers<br>Cancer Type:<br>Breast (75%)<br>Other (Colon, Kidney, Skin, or Non-Hodgkin Lymphoma) (25%)	- This study discussed the barriers to supportive cancer care experiences by First Nations survivors in four Indigenous communities.<br>- Survivors looked to their institution to provide financial aid for expensive medical travels, emotional and spiritual counseling for distress, and caregiver support.
Haq [52]<br>2013<br><br>Ontario	To (1) document information needs from the perspectives of breast cancer patients, FPs and oncology specialist health care providers (OHCPs); (2) design and implement an SCP based on identified needs; and (3) evaluate the pilot SCP's effectiveness at addressing these gaps.	*n* = 39<br>Breast (100%)<br>Age (at dx = 55.5 yr, range 35–85).	- Described a web-based SCP for breast cancer survivors, that addressed needs encountered at different phases (diagnosis, active treatment, and fu).<br>- There was a unanimous suggestion to provide the SCP earlier than EOT.<br>- Survivors expressed a desire for peer-support and a coordinator to assist them along their cancer trajectory.
Hodgson [57] (2010)	To evaluate the fu care received in an unselected, population-based cohort of	*n* = 2071<br>Hodgkin Lymphoma (100%)	- Most pts had visits with both the PCP and oncologist in yr 2–5 post-dx.

Ontario	Hodgkin Lymphoma (HL) survivors and, to determine the extent to which routine imaging, standard healthcare interventions, and HL-specific screening recommendations were used.	Age (mean = 35.4 yr, SD 17.06)	- Despite frequent MD contact, CT surveillance of pts may have been overused in during this time, whereas the rates of recommended cancer screening were suboptimal.
Jammu [23] 2020<br><br>Ontario	To determine the impact of the Cancer Survivorship Program intervention on patients' reported outcomes using distress thermometer (DT), Canadian Problem Checklist (CPC), and Edmonton Symptom Assessment Scale (ESAS).	*n* = 176<br>Breast (*n* = 149, 84.5%)<br>Other (*n* = 27, 15.5%)<br>Age<br><39 = 8,2.8%<br>40–49 = 23 (13.1%)<br>50–59 = 52 (29.6%)<br>60–69 = 57,32.4%)<br>70–79 = 31(17.6%)<br>>80 = 8 (4.5%)	- Described the Cancer Survivorship Program, a patient-centered care clinic to transition from cancer team to FP for pts who have completed ca tx and are in remission.<br>- The clinic provides inter-disciplinary care focusing on psychological, social, informational, emotional, practical support, and psychosocial recovery.<br>- Once pts are referred, an open communication channel is maintained with the referring oncology team.<br>- The clinic was found to decrease survivor distress, physical side effects, and depression/anxiety.
Jones [46] 2020<br><br>Eastern, Central and Western Canada	To describe the physical, emotional, and practical concerns in a large sample of Canadian AYA cancer survivors (ages 18–34 years) within the first 5 years post-treatment.	*n* = 575<br>Cancer Type<br>Hematological *n* = 139, 24.2% Thyroid *n* = 92, 16.0%<br>Testicular *n* = 77, 13.4%<br>Breast *n* = 49, 8.5%<br>Melanoma *n* = 47, 8.1%<br>Gynecological *n* = 39, 6.8%<br>Central nervous system *n* = 33, 5.7%<br>Sarcoma *n* = 24, 4.2%<br>Gastrointestinal *n* = 18, 3.1%<br>Other *n* = 41, 7.0%<br>Missing *n* = 20, 3.5%<br>Age<br>18–24 = 116 (20.2%)<br>25–29 = 213 (37.0%)<br>30–34 = 246 (42.8%)<br>Time since tx:<br><1 yr = 63 (11.0%);<br>1 to < 3 yr = 2999 (52.0%);<br>3 to 5 yr =170 (29.6%);<br>No tx received = 38 (6.6%);<br>Missing = 6 (1.0%))	- Young adult cancer survivors most frequently sought HCP help for physical changes.<br>- The most services reported to be accessible to cancer survivors were one-on-one counselling with a professional (28%), on-line peer support through social media (12%), and face-to-face peer support group (8%).<br>- Most often respondents were unaware of resources available to them.<br>- Many of the respondents reported using social media or accessing websites for cancer-related information and support.

Kwong [37]<br>2016<br><br>British Columbia	To help address unmet needs of Chinese-speaking cancer survivors, we have developed a structured psycho-educational program informed by both cultural values of the population and published recommendations for cancer survivorship education and support.	n = 123<br>Age (>50% were >60 yr)<br>Time since tx (most (57–69%) completed tx in the past 2 yr)	- A 6 week 2-hr program was developed by a multidisciplinary team using questions raised by a Chinese cancer support group: assess current post ca state; self-reflect and goal set; info, skills, and tools provided.<br>- Materials and inquiry phone line were bilingual.<br>- Participants were widely satisfied with the sessions and found them to be very informative.
Liska [22]<br>2018<br><br>Ontario	To describe the physical and psychosocial needs of colorectal and breast cancer survivors and their feelings of empowerment on entry into the Wellness Beyond Cancer Program (WBCP) and one year after transitioning to primary care.	n = 123<br>Colorectal [n = 53, 43%];<br>Breast [n = 70, 57%])<br>Age<br><49 yr = 8 CRC, 7 Breast<br>50–59 yr = 8 CRC, 18 Breast<br>60–69 yr = 12 CRC, 19 Breast<br>70–79 yr = 20 CRC, 17 Breast<br>>80 yr = 11 CRC, 9 Breast<br>Time since tx:<br><1 yr = 35 CRC, 13 Breast;<br>1–2 yr = 8 CRC, 7 Breast<br>2–3 yr = 5 CRC, 7 Breast<br>5–10 yr = 1 CRC, 25 Breast<br>>10 yr = 1 CRC, 5 Breast	- Survivors participated in WBCP which was composed of a 2-hr educational session, disease-specific print and web-based resources, and an individualized SCP prepared and delivered by an oncology nurse.<br>- SCP outlined the pt's ca care team, disease and tx summary, and fu guidelines.<br>- Patients' self-identified physical and psychosocial needs that were indicated as being moderate to extreme on the needs assessment were included in the care plan.<br>- The WBCP phone number was also provided to the PCP to address potential future questions/concerns and to ensure an expedited re-entry to the cancer program, if necessary.<br>- All participants felt empowered by participating in the program.<br>- There were no significant impacts on CRC survivors' physical or psychosocial needs, apart from fear of cancer recurrence, which increased.<br>- Breast cancer survivors had no statistically significant changes over time.
McBride [24]<br>2019<br><br>British Columbia, Manitoba, Ontario, Nova Scotia	To assess and compare proportions of pts in each province who received adherent and non-adherent surveillance for recurrence, new ca and late effects, recommended preventive care, and recommended physician visits for comorbidities.	n = 38461<br>(Breast 100%)<br>BC = 9338; (Age—median at dx = 60 yr (IQR 51–70)).<br>MB = 2688; (Age—median at dx = 61 yr (IQR 51–70)).<br>ON = 23,700; (Age—median at dx = 60 yr (IQR 50–69)).<br>NS = 2735; (Age—median at dx = 61 yr (IQR 51–70))	- For all provinces and in all fu yr, most pts had more than the recommended number of visits to oncologists or PCPs.<br>- For all provinces, most pts in each fu yr received guideline-based surveillance imaging; MB and ON had higher adherence than BC and NS.<br>- Compliance with recommended physician visits for pts with several chronic conditions was high in ON and NS; lower levels of compliance were observed in BC.<br>- Preventive care (e.g., cervical/colon ca screening, bone densitometry) was less than optimal in all provinces with available data.

Miedema [41] 2013<br><br>Eastern, Central and Western Canada	To assess whether current cancer follow-up care practices meet the needs of young adult cancer survivors in Canada.	*n* = 53.<br>Cancer Type:<br>Breast (*n* = 13, 25%)<br>Thyroid (*n* = 12, 23%)<br>Non-Hodgkin Lymphoma (*n* = 8, 15%)<br>Hodgkin Lymphoma (*n* = 6, 11%)<br>Testicular (*n* = 4, 7%)<br>Other (*n* = 10, 19%)<br>Age (mean = 32 yr,SD 6.43, range 18–39)<br>Time since dx: yr = 1(2%);<br>1 yr = 17(32%);<br>2 yr = 9(17%);<br>3 yr = 6(11%);<br>4 yr = 6(11%);<br>5 yr = 8(15%);<br>>5 yr = 6(11%)	- Over 70% of participants reported fertility as an issue, but fertility preservation (e.g., sperm banking) was attempted by only a few participants.<br>- Twenty-one percent of participants lacked supplemental health insurance and so had to personally pay for all of their medication and allied health care services.
Mitchell [27] 2007<br><br>Ontario	To investigate the psychosocial impact of dragon boat participation on women who have been treated for breast cancer.	*n* = 10<br>Breast (100%)<br>Age(range = 35–70 yr)<br>Time since dx (range 1–4 yr)	- Women sought emotional, social, and physical benefits from becoming a member of a Dragon Boat team in a community with other breast cancer survivors.<br>- Survivors reported participating as a means of increasing their physical activity and ended up experiencing an extraordinary level of support from their teammates.
Norris [30] 2015<br><br>Alberta	To estimate the potential magnitude of any additional benefits of 3 versus 2 days per week of resistance training (RT) in prostate cancer survivors.	*n* = 30<br>Prostate (100%)<br>Age (mean = 63 yr, range 46–76)<br>Time since dx = 14 mo (range 2–22);<br>completed <12 mo = 15 (50%)	- Described a 12-wk resistance training program, either 2 or 3 days per wk, with at least one rest day between sessions.<br>- Each session contained warm up, full body resistance training, and a cool down.<br>- Both groups experienced improved general health, muscular strength, mental health, and a significant improvement in prostate cancer symptoms.
Peddle [28] 2008<br><br>Alberta	To examine quality of life and fatigue in colorectal cancer survivors meeting and not meeting public health exercise guidelines.	*n* = 413<br>Cancer Type<br>Colon = 318 [77%];<br>Rectal = 95 [23%])<br>Age (mean = 60.7 yr, SD 7.4)<br>Time since dx:<br><60 mos = 250 (60.5%)<br>≥60 mos = 163 (39.5%)	- Pts were categorized as meeting or not meeting exercise guidelines.<br>- Results indicated the changing trajectory of exercise from pre-dx, while on tx, and post-tx.<br>- Pts meeting exercise guidelines of >150 min per week had the highest quality of life and least fatigue.
Railton [18] 2015<br>Alberta	To examine adherence with follow-up guidelines, symptoms, and need for a telephone-based survivorship clinic.	*n* = 240<br>Breast (100%)<br>Age at dx=<br><50 yr (*n* = 112, 46.7%);<br>≥50 yr (*n* = 128, 53.3%))	- Adherence to guideline recommendations was high for mammography and exams.<br>- Oncologists provided endocrine therapy plan to 97.5% of pts who had hormone-responsive cancer and adherence to endocrine therapy was high (82.3%),

		Time from dx<br>12–24 mos (n = 11, 4.6%);<br>25–36 mos (n = 61, 25.4%);<br>37–48 mos (n = 89, 37.1%);<br>>48 mos (n = 79, 32.9%))	although 22.7% of pts had nonadherent and nonpersistent use.
Saroa [45]<br>2018<br><br>Alberta	To understand the information needs and information source preferences of individuals aged 18–65 years at the time of diagnosis and who were in the post-tx phase of recovery following dx with HNC tumors consistently associated with HPV.	n = 203<br>Head and neck ca (100%).<br>Age (mean = 58.3 yr, SD 6.5, range = 35–71)<br>Time since tx (mean = 28 mo, SD 17.9, range = 2–61)	- Most used sources of info (in descending order) were HCPs, family and friends, the Internet, and written materials.<br>- The use of HCPs as an information source increased 1.012x for each mo since tx.<br>- Using written materials as an information source was 61% less common in men than women.<br>- The use of television or family and friends as information sources increased 2.2x and 1.96x, respectively, with each level of education.
Shannon [44]<br>2005<br><br>New Brunswick	To explore the role and meaning of leisure and social support in the lives of women who completed tx for breast ca >12m and were part of a formal support network.	n = 8.<br>Breast (100%)<br>Age (range = 36–55 yr)<br>Time since tx (6 yr = 3; 4 yr = 2; 3 yr = 2; 1 yr = 1)	- Survivors relied on their family, friends, co-workers, and neighbours for tangible support such as household tasks (e.g., bringing food, or assisting with childcare, household tasks, and transportation).<br>- Friends and family were a source of emotional support.<br>- Volunteering and support groups were a form of social support.
Singh-Carlson [17]<br>2018<br><br>British Columbia	To better understand the development of a survivorship care plan (SCP) and its delivery and utility in the population of interest. To confirm the content and format of the SCP within the population of interest.	n = 16<br>Breast (100%)<br>Age (mean = 55, range 32–66)	- 100% of survivors found the SCP to be useful in providing information about physical effects after tx and resources.<br>- Participants reported not feeling confident in their FP's ability to manage their psychosocial symptoms or hormone therapy.<br>- Family, faith, and FP were the top three supportive resources used at 1-yr post-tx.
Sisler [19]<br>2012<br><br>Manitoba	To examine how colorectal cancer (CRC) survivors evaluate the continuity and quality of their cancer follow-up care, particularly in those who identify a PCP as the main provider of that fu.	n = 246<br>Cancer Type<br>Colon = 153 [62.2%]<br>Rectum = 93 [37.8%]<br>Age (mean = 70 yr)<br>Time since dx (mean =18 mo,range 13–24)	- 47% pts reported PCP or oncologist as responsible for their fu care; the other 53% indicated multiple providers.<br>- Those who named a PCP as a main provider, reported a high level of satisfaction with continuity of care and felt prepared to have their care transferred to the PCP from the cancer clinic.
Tracy [36]<br>2016<br><br>British Columbia	To pilot test a Sexual Health Clinic (SHC) for cancer survivors at a major Canadian cancer treatment centre to determine the feasibility and effectiveness.	n = 21<br>11 male [mean age = 66.6 yr, ±4.3]<br>10 female [mean age = 56.6 yr ± 9.5]<br>Cancer Type:<br>Prostate n = 8, 38%<br>Colorectal n = 2, 10%	- Cancer survivors reporting significant changes to their sexual health attended a SHC where they received assessment, education, and tailored sexual health support by an oncology nurse with specialized skills in sexual health.<br>- Pts engaged with the SHC for solutions to problematic changes to their sexual health (e.g., vaginal

		Breast n = 7, 33%<br>Endometrial n = 2, 10%<br>Lung n = 1, 5%<br>Lymphoma n = 1, 5%	dryness, erectile dysfunction, loss of libido, etc.) which resulted from cancer and/or cancer tx.<br>- Sixteen pts reported overall improvement in their wellbeing and sexual life after attending the SHC.
Trinh [34]<br>2018<br><br>Ontario	To examine feasibility (e.g., recruitment, adherence, number of adverse events) and acceptability of a web-based intervention (RiseTx) for reducing sedentary behaviour (SED) and increasing mod-to-vig physical activity (MVPA) among prostate cancer survivors (PCS) undergoing ADT.	n = 42<br>Prostate (100%)<br>Age (mean= 73.2 yr, SD± 7.3)<br>Time since dx (mean = 93.6 mo, SD 71.2)	- The web-based intervention consisted of five phases to increase physical activity.<br>- Following the program, SED decreased and MVPA increased right after intervention but no significant effect on QOL were seen.<br>- Pts were satisfied with the intervention, but additional strategies may be needed for maintenance of behavior change.
Urquart [20]<br>2017<br><br>Nova Scotia	To investigate patterns of routine fu care at a cancer centre for breast, colorectal, gynecologic, and prostate ca survivors; factors associated with receipt of fu care at a cancer centre; and changes in follow-up care at a cancer centre over time.	n = 12,267<br>pts who had 1+ visit at the cancer centre for consult or tx.	- Respectively, 50.1%, 30.2%, 47.2%, and 62.4% of breast, colorectal, gynecologic, and prostate ca survivors had ≥1 fu visit at a cancer centre.<br>- 19.2% of the survivors had ≥5 routine fu visits at a cancer centre, with that proportion also varying by disease site (21.2%, 10.3%, 26.8%, and 19.9% of breast, colorectal, gynecologic, and prostate cancer survivors, respectively).<br>- Controlling for other factors, sex, and residential status (distance to closest cancer centre, rural or urban residence) had no effect on the likelihood of fu visits at the cancer centre.
Vallance [29]<br>2007<br><br>Alberta	To determine the effects of breast cancer-specific print materials and step pedometers on physical activity (PA) and quality of life (QoL) in breast ca survivors.	n = 377<br>Breast (100%)<br>Age (mean = 58 yr,range 30–90).<br>Time since dx (mean = 39 mo, SD 11.3).	- Survivors in a physical activity program using pedometers were found to have increased physical activity and quality of life, and decreased levels of fatigue.
Wiljer [26]<br>2013<br><br>Ontario	To explore the survivor experience and impact of the survivorship consult (SC) in order to improve the usefulness and effectiveness of the SC in a clinical setting.	n = 25<br>Breast (100%)<br>Age<br>35–44 yr n = 5, 19.2%<br>45–54 yr n = 6, 23.1%<br>55–64 yr n = 11, 42.3%<br>65+ yr n = 3, 11.5%	- Described a "survivorship consult" program designed to help survivors reflect on their dx, tx, and strategies for coping, and to address their needs for info, education, and support.

Abbreviations: dx—diagnosis; IQR—interquartile range; FP—family practitioner/physician; fu—follow-up; PCP primary care provider/professional; QOL—quality of life; mo—month(s); yr—years; wk—week.

Table S3. Process for coding and combining cancer survivorship recommendations by CCO [4] and CAPO/CPAC [6].

Combined Recommendation Used in Table 3	CCO Recommendations	CAPO/CPAC Recommendations

**Specialist to inform patient about transition to PCP**	**CCO-1:** One of the specialist teams (i.e., surgical, radiation, or systemic) should inform the individual that they may be transitioned back to their primary care provider for ongoing follow-up after their treatment is completed.	
**Specialist to assume responsibility for follow-up planning prior to end of treatment**	**CCO-2**: If the individual receives more than one modality of treatment (e.g., surgery, radiation, and/or systemic treatment), one of these specialist teams should assume the responsibility for follow-up care planning near the completion of the treatment.	**A-4:** It is recommended that one or more health care providers be designated as responsible for providing survivorship follow-up services, with integration of primary care physicians in monitoring for late and long-term treatment consequences, coordinated access to interdisciplinary specialists as required, with an emphasis on actively engaging and empowering survivors.
**Co-creation of individualized follow-up care plan**	**CCO-3:** Close to the end of treatment, the specialist team responsible for follow-up care planning should work with the survivor and all relevant care teams (e.g., surgical, radiation, and/or systemic) to develop a follow-up care plan.	**A-2:** It is recommended that individuals completing cancer treatment and their families receive individualized information and support in consultation with a designated and skilled member of the health care team to prepare them for the life-long monitoring and follow-up care required post-cancer treatment, and to minimize distress in the transition from active treatment to the follow-up phase of the cancer journey.
**Treatment summary, follow-up plan and contacts given to patient**	**CCO-4:** The survivor should have a follow-up care plan that is comprised of two components: a) a treatment summary (at minimum a set of key clinical information that includes, but is not limited to, cancer diagnosis, treatment received, and a reconciled list of medications) b) an individualized plan of care for follow-up based on the needs of the survivor (at guidelines/recommendations for follow-up care, a list of signs and symptoms of recurrence, long-term, and late effects, and psychosocial needs). Additionally, instructions for referral or re-entry into the cancer system, contact information of the treatment team who prepared the individualized plan of care, and the provider who will be responsible for ongoing follow-up care should be provided.	**A-3:** It is recommended that all individuals completing primary treatment for cancer receive a written treatment summary and follow-up care plan (Survivorship Care Plan) from a designated member of the care team that includes a standard set of core multidimensional elements tailored to the individual's cancer and treatment experience. (Recommendation adapted from IOM consensus recommendation #2).
**MRP designated, and their contact details known to patient**	**CCO-5:** The survivor should have a most responsible provider (MRP) for ongoing follow-up care. The name and contact information of the MRP should be documented in the follow-up care plan. The MRP may be a primary care provider or a specialist.	**A-4:** It is recommended that one or more health care providers be designated as responsible for providing survivorship follow-up services, with integration of primary care physicians in monitoring for late and long-term treatment consequences, coordinated access to interdisciplinary specialists as required, with an emphasis on actively engaging and empowering survivors.
**All care teams receive**	**CCO-6:** All care teams involved in the survivor's follow-up care should receive a copy of the follow-up care plan.	

**follow-up care plan**		
**Pts receive and review follow-up care plan with MRP**	**CCO-7:** The survivor should receive a copy of the follow-up care plan and review it with the MRP and other members of the care team to ensure that the survivor understands the care plan and its goals.	**A-2:** It is recommended that individuals completing cancer treatment and their families receive individualized information and support in consultation with a designated and skilled member of the health care team to prepare them for the life-long monitoring and follow-up care required post-cancer treatment, and to minimize distress in the transition from active treatment to the follow-up phase of the cancer journey.
**MRP coordinate surveillance tests well in advance**	**CCO-8:** The MRP for follow-up care should be responsible for ordering and coordinating surveillance tests for the survivor, including those who are receiving adjuvant hormonal therapies. The surveillance tests should be ordered well in advance to ensure that they are performed in accordance with the interval as recommended by evidence-based clinical guidance.	**A-4:** It is recommended that one or more health care providers be designated as responsible for providing survivorship follow-up services, with integration of primary care physicians in monitoring for late and long-term treatment consequences, coordinated access to interdisciplinary specialists as required, with an emphasis on actively engaging and empowering survivors.
**PCP as MRP has timely access to specialist consultation**	**CCO-9**: If the MRP is a primary care provider, they should have timely access to specialist consultation in cases of uncertainty related to suspicion of cancer recurrence, metastasis, or second cancers.	**A-4:** It is recommended that one or more health care providers be designated as responsible for providing survivorship follow-up services, with integration of primary care physicians in monitoring for late and long-term treatment consequences, coordinated access to interdisciplinary specialists as required, with an emphasis on actively engaging and empowering survivors.
**Streamlined re-entry process into the cancer system**	**CCO-10:** A streamlined process should be available for the MRP to arrange for re-entry of the survivor into the cancer system in case of suspicion.	
**Appropriate sharing of surveillance test results with the patient**	**CCO-11:** The MRP for follow-up care should offer in-person or secure telehealth options (e.g., phone call, videoconference, online patient health information applications), when appropriate, to share surveillance test results with the survivor.	
**Regular assessment and/or referral of physical and psychosocial needs including:**<br>- **Screening for distress**<br>- **Managing**	**CCO-12:** The MRP for follow-up care should regularly assess any medical and psychosocial late and long-term effects and arrange for appropriate services. The MRP should also assess caregiver distress and, if needed, coordinate supports for caregivers.	**A-1:** It is recommended that survivorship services be recognized as a distinct component and standard of cancer care, with access to services to meet a broad range of survivors' physical, psychosocial, supportive, informational, and rehabilitative needs. (Recommendation adapted from the Institute of Medicine's (IOM) consensus recommendation #2).<br><br>**A-5**: It is recommended that survivors be routinely screened for distress using valid tools across a broad range of late and long-term treatment effects: persistent symptoms and functional problems, symptoms of mood disorders (anxiety and depression), and other common problems such as

**psychosocial concerns & distress**<br>- **Monitoring symptoms & late/long-term effects (b**<br>- **Managing sexual health concerns**<br>- **Managing post treatment fatigue**<br>- **Managing vasomotor symptoms**<br>- **Managing sleep-wake disruptions**		cognitive changes or alterations in sexual health. Screening should be followed by focused assessment and interventions based on recommendations found in evidence-based clinical practice guidelines. (Recommendation adapted from IOM consensus recommendation #3, and Psychosocial Health Care Needs Assessment Guideline for Adults, 2009).<br><br>**B-3:** It is recommended that survivors at risk of, or with identified and significant, psychosocial concerns or distress be offered referral to psychosocial health services, individualized or group-based cognitive behavioural or psycho-educational programs provided by trained professionals.<br><br>**B-4:** It is recommended that protocols for routine follow-ups include monitoring for and managing physiological and psychosocial symptoms, including pain and fatigue, and late and long-term effects, such as pulmonary or cardiac effects, osteoporosis, and other endocrine or body system abnormalities. A coordinated shared-care approach should be used, including referrals to appropriate interdisciplinary team members as appropriate.<br><br>**B-5:** It is recommended that survivors receive specific psycho-educational-based care regarding changes in sexual health and function. They should have access to programs that include couple's therapy for both the cancer survivor and his or her partner, and sexual rehabilitation programs to promote healthy post-treatment sexual health and maximize function.<br><br>**B-6:** It is recommended that survivors be screened for cancer-related fatigue and have access to exercise programs combined with psychoeducational interventions and/or multi-component cognitive behavioural therapy to manage post-treatment fatigue.<br><br>**B-7:** It is recommended that all female cancer survivors have access to multi-component cognitive behavioural therapy and lifestyle management programs to effectively manage vasomotor symptoms. This is also important for other cancer survivors, such as those with prostate cancer, where hormonal deprivation therapies may lead to significant physical and emotional effects.<br><br>**B-8:** It is recommended that survivors have access to multi-component cognitive behavioural therapy programs to manage disruptions in sleep–wake patterns.
**PCP manage side effects, provide supportive care and self-**	**CCO-13:** Primary care providers should manage side effects, long-term and late effects experienced by the survivor and provide supportive care and self-management support, when appropriate.	**A-6:** It is recommended that using approaches recommended for supporting effective self-management, designated providers of survivorship follow-up care should focus on enabling and empowering individuals and their families by

**management support**<br>- **Support survivor engagement & self-management,**<br>- **Support healthy behaviours**		giving them the skills and knowledge they need to be active participants in optimizing their health and wellbeing.<br><br>**B-1:** It is recommended that survivors have access to self-management focused education and support to facilitate the tailored adoption of healthy lifestyle behaviours inclusive of: daily physical activity; balanced nutrition; and smoking cessation programs designed to improve health-related quality-of-life and physiological outcomes, and reduce distress and risk of recurrence.
**Variable delivery of supportive care and info**	**CCO-14:** Various delivery strategies should be considered when providing supportive care and information to the survivor. These strategies may include, but are not limited to, consultation by phone, referral to credible online and/or community resources (e.g., psychosocial oncology programs at cancer centres, community or online-based cancer support programs), and group-based educational sessions.	
**Self-management and quality resources for patients**	**CCO-15:** The survivor should have access to structured self-management programs and high-quality resources that can inform and educate them on how to manage their own physical and psychosocial symptoms (e.g., fatigue, pain). Self-management strategies should be discussed with the survivor prior to completion of the treatment and during their follow-up visits.	**A-6:** It is recommended that using approaches recommended for supporting effective self-management, designated providers of survivorship follow-up care should focus on enabling and empowering individuals and their families by giving them the skills and knowledge they need to be active participants in optimizing their health and wellbeing.<br><br>**B-1:** It is recommended that survivors have access to self-management focused education and support to facilitate the tailored adoption of healthy lifestyle behaviours inclusive of: daily physical activity; balanced nutrition; and smoking cessation programs designed to improve health related quality-of-life and physiological outcomes, and reduce distress and risk of recurrence.
**Clinical staff education**		**A-7:** It is recommended that all clinical staff receive education to increase awareness of the needs of cancer survivors. Specific education programs should be targeted to designated follow-up care providers to ensure effective monitoring for disease recurrence, preventing and managing late and long-term effects of cancer treatment, and to encourage specific strategies that empower survivors to be actively engaged in self-management and adopt healthy lifestyle behaviours.
**Programs based on behaviour change theories**		**B-2:** It is recommended that psychosocial and supportive care programs and interventions be designed based on health-behaviour change theories that are known to be influential and necessary for sustaining the adoption of healthy lifestyle behaviours.

Table S4. A mapping of cancer follow-up recommendations [4,6] reflected in the reviewed papers.

Author Year	Alimujiang [49] 2019	Bender [14] 2016	Bilodeau[42] 2019	Boekhout [55] 2015	Bowler [53] 2019	Brunet [32] 2016	Chahine [54] 2019	Chongya [50] 2015	Collie [51] 2014	Cyr [56]2020	Dawe [43] 2014	Easley [39] 2012	Farris [33] 2017	Fillion [35] 2008	Galica [38] 2020	Gall [48] 2004	Gaudine [25] 2003	Gifford [47] 2019	Goldenberg [21] 2020	Grant [15] 2015	Hammond [40] 2017	Haq [52] 2013	Hodgson [57] 2010	Jammu [23] 2020	Jones [46] 2020	Kwong [37] 2016	Liska [22] 2018	Mcbride [24] 2019	Miedema [41] 2013	Mitchell [27] 2007	Norris [30] 2015	Peddle [28] 2008	Railton [18] 2015	Saroa [45] 2018	Shannon [44] 2005	Singh-Carlson [17] 2018	Sisler [19] 2012	Tracy [36] 2016	Trinh [34] 2018	Urquhart [20] 2017	Vallance [29] 2007	Wiljer [26] 2013
Specialist inform pt about transition to PCP (CCO1)	**-**	**-**	**-**	**Y**	**-**	**-**	**-**	**-**	**-**	**-**	**-**	**-**	**-**	**-**	**-**	**-**	**-**	**-**	**Y**	**Y**	**-**	**-**	**-**	**Y**	**-**	**-**	**Y**	**-**	**-**	**-**	**-**	**-**	**-**	**-**	**-**	**Y**	**-**	**-**	**-**	**-**	**-**	**-**
Specialist assume responsibility for fu planning prior to eot (CCO2 & A-4)	-	-	-	-	-	-	-	-	-	-	-	-	-	-	-	-	-	-	Y	Y	-	-	-	-	-	-	Y	-	-	-	-	-	-	-	-	-	-	-	-	-	-	-
Co-creation of individualized fu care plan (CCO3 & A-2)	-	-	-	-	-	-	-	-	Y	-	-	-	-	-	-	-	-	-	P	-	-	-	-	Y	-	-	Y	-	-	-	-	-	-	-	-	Y	-	-	-	-	-	Y
Treatment summary [A], fu plan [B] and contacts[C] given to pt (CCO4 & A-3)	-	-	-	Y	P[A,B]	-	-	-	Y	-	-	-	-	-	-	-	-	-	P A.B	Y	-	Y	-	-	-	-	Y	-	-	-	-	-	P[A]	-	-	Y	-	-	-	-	-	P A,B
MRP designated, and their contact details known to pt (CCO5 & A-4)	-	-	-	Y	Y	-	-	-	Y	-	-	-	-	-	-	-	-	-	-	Y	-	-	-	Y	-	-	Y	-	-	-	-	-	-	-	-	-	-	-	-	-	-	-
All care teams receive fu care plan (CCO6)	-	-	-	Y	-	-	-	-	Y	-	-	-	-	-	-	-	-	-	Y	Y	-	Y	-	Y	-	-	Y	-	-	-	-	-	-	-	-	Y	-	-	-	-	-	-
Pt receive[D] and review[E] fu care plan with MRP (CCO7 & A-2)	-	-	-	Y	-	-	P [D]	-	Y	-	-	-	-	-	-	-	-	-	Y	P [D]	-	-	-	-	-	-	Y	-	-	-	-	-	-	-	-	Y	-	-	-	-	-	Y
MRP coordinate surveillance tests well in advance (CCO8 & A-4)	-	-	-	-	-	-	-	-	Y	-	-	-	-	-	-	-	-	-	Y	-	-	-	Y	Y	-	-	-	Y	-	-	-	-	-	-	-	-	-	-	-	-	-	-
PCP as MRP has	-	-	-	-	-	-	-	-	-	-	-	-	-	-	-	-	-	-	-	Y	-	-	-	Y	-	-	-	-	-	-	-	-	-	-	-	-	-	-	-	-	-	-

timely access to specialist consultation (CCO9 & A-4)																																										
Streamlined re-entry process into cancer system (CCO10)	-	-	-	-	-	-	-	-	-	-	-	-	-	-	-	-	-	-	-	Y	-	-	-	Y	-	-	Y	-	-	-	-	-	-	-	-	-	-	-	-	-	-	Y
Appropriate sharing of surveillance test results with pt (CCO11)	-	-	-	-	-	-	-	-	-	-	-	-	-	-	-	-	-	-	-	-	-	-	-	-	-	-	-	-	-	-	-	-	-	-	-	-	-	-	-	-	-	-
Regular assessment[F] and/or referral[G] of physical[H]-psycho-social[I] needs (CCO12 & A-1)	-	-	-	-	-	-	-	-	-	-	-	-	-	-	-	-	-	-	Y	-	-	-	P[F,H]	Y	-	-	-	P[F,H]	-	-	-	-	-	-	-	-	-	P[F,G,H]	-	-	-	-
Screening for distress (A-5)	-	-	-	-	-	-	-	-	Y	-	-	-	-	-	-	-	-	-	-	-	-	-	-	Y	-	-	-	-	-	-	-	-	-	-	-	-	-	-	-	-	-	-
Manage psychosocial concerns & distress (B-3)	-	-	-	-	Y	-	-	-	Y	-	-	-	-	-	-	-	-	-	Y	-	-	-	-	Y	-	-	-	-	-	-	-	-	-	-	-	-	-	-	-	-	-	-
Monitoring symptoms & late/long-term effects (B-4)	-	-	-	-	Y	-	-	-	Y	-	-	-	-	-	-	-	-	-	-	Y	-	-	-	Y	-	-	-	-	-	-	-	-	-	-	-	-	-	-	-	-	-	-
Managing sexual health concerns (B-5)	-	-	-	-	Y	-	-	-	Y	Y	-	-	-	-	-	-	-	-	-	-	-	-	-	-	-	-	-	-	-	-	-	-	-	-	-	Y	-	Y	-	-	-	-
Managing post-treatment fatigue (B-6)	-	-	-	-	Y	-	-	-	-	-	-	-	-	Y	-	-	-	-	-	-	-	-	-	-	-	-	-	-	-	-	-	Y	-	-	-	-	-	-	-	-	Y	-
Managing vasomotor symptoms (B-7)	-	-	-	-	-	-	-	-	-	-	-	-	-	-	-	-	-	-	-	-	-	-	-	-	-	-	-	-	-	-	-	-	-	-	-	-	-	-	-	-	-	-
Managing sleep–wake disruptions (B-8)	-	-	-	-	Y	-	-	-	-	-	-	-	-	-	-	-	-	-	Y	-	-	-	-	-	-	-	-	-	-	-	-	-	-	-	-	-	-	-	-	-	-	-
PCP manage side effects, provide supportive care and self-managemen	-	-	-	-	-	-	-	-	-	-	Y	-	-	-	-	-	-	-	-	-	-	Y	-	Y	-	-	-	-	-	-	-	-	-	-	-	-	-	-	-	-	-	-

t support (CCO13)																																										
Support survivor engagement [J] & self-management [K] (A-6)	-	-	-	-	-	Y	-	-	-	-	-	-	-	-	-	-	-	-	-	-	-	Y	-	-	-	-	-	-	-	-	P [K]	-	-	-	-	-	-	-	P [J]	-	P [J]	Y
Support healthy behaviours (B-1)	-	-	-	-	Y	-	-	Y	-	-	-	-	-	-	-	-	-	-	-	-	-	Y	-	Y	-	Y	-	-	-	-	Y	Y	-	-	-	Y	-	-	Y	-	Y	-
Variable delivery of supportive care and info (CCO14)	-	Y	-	-	-	-	-	-	-	Y	-	-	-	-	Y	-	-	-	-	-	-	Y	Y	-	Y	Y	Y	-	-	Y	-	-	-	Y	-	-	-	-	-	-	-	-
Self-management [L] and quality resources [M] for pts (CCO15 & A-6, B-1)	P [L]	-	-	-	Y	P [M]	-	-	Y	P [L,M]	-	-	-	-	-	-	-	-	Y	Y	-	-	-	P [M]	P [M]	Y	Y	-	-	-	P [M]	-	-	-	-	-	-	P [M]	Y	-	P [M]	Y
Clinical staff education (A-7)	-	-	-	-	-	-	-	-	-	Y	-	-	-	-	-	-	-	-	-	-	-	Y	-	-	-	-	-	-	-	-	-	-	-	-	-	-	-	-	-	-	-	Y
Programs based on behaviour change theories (B-2)	-	-	-	-	-	-	-	-	-	-	-	-	-	-	-	-	-	-	-	-	-	-	-	-	-	-	-	-	-	-	-	-	-	-	-	-	-	-	-	-	-	-

Y—content included; P—partially included (portion of the element included denoted by supra script letter); - —not indicated/unclear.
